# Exploring Transduction Mechanisms of Protein Transduction Domains (PTDs) in Living Cells Utilizing Single-Quantum Dot Tracking (SQT) Technology

**DOI:** 10.3390/s120100549

**Published:** 2012-01-05

**Authors:** Yasuhiro Suzuki

**Affiliations:** Department of Emerging Infectious Diseases, Graduate School of Medicine, Tohoku University, Seiryo-machi, Aoba-ku, Sendai 980-8575, Japan; E-Mail: suzukiy39@med.tohoku.ac.jp; Tel: +81-022-717-8220; Fax: +81-022-717-8221

**Keywords:** protein transduction domain, HIV-1, Tat, quantum dot, single particle tracking

## Abstract

Specific protein domains known as protein transduction domains (PTDs) can permeate cell membranes and deliver proteins or bioactive materials into living cells. Various approaches have been applied for improving their transduction efficacy. It is, therefore, crucial to clarify the entry mechanisms and to identify the rate-limiting steps. Because of technical limitations for imaging PTD behavior on cells with conventional fluorescent-dyes, how PTDs enter the cells has been a topic of much debate. Utilizing quantum dots (QDs), we recently tracked the behavior of PTD that was derived from HIV-1 Tat (TatP) in living cells at the single-molecule level with 7-nm special precision. In this review article, we initially summarize the controversy on TatP entry mechanisms; thereafter, we will focus on our recent findings on single-TatP-QD tracking (SQT), to identify the major sequential steps of intracellular delivery in living cells and to discuss how SQT can easily provide direct information on TatP entry mechanisms. As a primer for SQT study, we also discuss the latest findings on single particle tracking of various molecules on the plasma membrane. Finally, we discuss the problems of QDs and the challenges for the future in utilizing currently available QD probes for SQT. In conclusion, direct identification of the rate-limiting steps of PTD entry with SQT should dramatically improve the methods for enhancing transduction efficiency.

## 1. Introduction

It has been believed that peptides and proteins usually do not penetrate the cell membrane. Therefore, to make proteins/peptides function inside the cell, viral vector-mediated gene expression or gene transfection are considered as the most reliable approach for expressing functional proteins/peptides *de novo* in cells. An alternative approach that appears to be the safest is to produce recombinant proteins exogenously and then to inject them into individual target cells by micro-injectors. However, there are several drawbacks to these methods; for example, the efficiency of gene introduction by viral vector-mediated methods is not adequate for non-dividing cells. On the other hand, owing to the complexity of the procedure, localized injections cannot handle many cells at a time. Furthermore, to analyze the function of a gene at the individual level, a large time- and labor-consuming effort is required for generating genetically modified organisms, such as mouse, fly, or nematodes. Instead of these methods, direct protein transduction and the functioning in cells or tissues of a protein transduction domain has received substantial attention recently. This approach appears to be excellent with respect to safety because this technique does not alter the host genome in comparison to gene expression methods. Moreover, it is expected that researchers will accelerate their speed for analyzing the function of proteins on both cellular and tissue levels; thus, protein transduction is one of the most promising technologies in the post-genomic era but research on this method is not yet complete.

Because a cell is isolated from the extracellular environment by the lipid bilayer plasma membrane, it was thought to be difficult to directly introduce proteins into the cells from outside. However, in 1988, Frankel and Pabo [1] and Green and Lowenstein [2] independently demonstrated that extracellular human immunodeficiency virus I type (HIV-1) trans-activator of transcription protein (Tat) crosses the plasma cell membranes and enters living cells. Thereafter, proteins with direct protein transduction ability were found in a wide variety of species (Table 1), such as antennapedia, a Drosophila homeodomain transcription factor [3], and VP22, a Herpes simplex virus 1 structural protein [4]. Subsequently, a more detailed analysis of the proteins led to the identification of a 10 to 20 amino acid sequence that has been designated as the Protein Transduction Domain (PTD). This segment has been found to be essential for transducing the proteins [5–10]. The group of amino acids that are basic in nature, including Arg and Lys, is critical in the PTD, to allow the proteins to cross the cell membranes. Several studies have reported that gene engineering or chemical modification, which can fuse or conjugate the target protein to a PTD, show protein transduction properties *in vivo* and *in vitro* [5,11–15]. These results accelerate the critical research questions to be answered for utilizing the PTD for transducing the target protein. They have also been shown to mediate the efficient intracellular accumulation of various biologically active substances such as antisense oligonucleotides [16], plasmids [8,17,18], peptide nucleic acids [19,20], short interfering RNAs (siRNAs) [21], radioisotopes [22], iron nanoparticles [23], and liposomes [24]. They can also effectively suppress the functions of signaling molecules [25,26] or transcription factors [27] by introducing synthetic peptides that can inhibit enzymatic activity. Moreover, it has been speculated that they open up a new avenue for introducing various molecules, such as fluorophores, for labeling intracellular molecules [28,29], and affinity labeling agents to analyze the intracellular-molecular interactions in living cells.

In summary, protein transduction methods have the following advantages: (i) transduction procedures are easy, and the effects can be observed in a short period; (ii) not only the protein but also various macromolecules can be introduced into the cells; (iii) they are very safe, as they do not alter the host genome; and (iv) bioactive macromolecules can be introduced into tissues by simple intravenous or intraperitoneal injection. Therefore, protein transduction methods are expected to be an innovative technology for improving the efficacy of delivering bioactive molecules as well as quickly analyzing the protein function both *in vitro* and *in vivo*.

However, protein transduction technologies have some important limitations [30,31]. For example, transduction efficiency is greatly influenced by the character of the target protein and, in many cases, sufficient efficacies were rarely achieved even with *in vivo* experiments [31]. To overcome these problems, extensive research is currently being focused on improving the transduction efficacy. In contrast, the pathway by which these peptides enter the cells has been the subject of substantial controversy over the last decade. This controversy was partly due to the lack of state-of-the-art techniques for the direct visualization of the behavior of PTDs; thus, the initial events at the cell surface have been predicted based only on biological activities (e.g., the activity of the reporter genes) or the amount of PTDs or PTD-fused protein that is inside the cells after 1 h of exposure. As a result, improving the efficacy, such as identifying the PTD derivative peptides that have a higher transduction efficiency, still depends on the biological activities measured by black boxing the transduction machinery.

In the present review, we initially focus on the outcome of using quantum dot (QD) labeled HIV-1-Tat PTD (TatP), which is the most intensely studied yet the least understood peptide in protein transduction. We focus also on the visualization of single particles and their entry mechanisms. In this regard, we share our recent findings using single QD imaging of TatP in living cells, by virtue of replicating this method, and using QD probes to explore novel findings in many other biological events. Therefore, we first discuss the currently proposed models of protein transduction that focus mainly on the TatP. Then, as a primer for a single particle study, we discuss the latest findings that were obtained by single particle tracking of various molecules on the plasma membrane [32]. Thereafter, we will introduce our findings, and finally, we will discuss the problems of using currently available QD probes for live cell imaging.

## 2. Controversies in Protein Transduction Mechanisms and the Ambiguities that Arise from Conventional Dyes

PTDs have been shown to be structurally diverse and highly variable in character. Their common feature is the existence of basic amino acid residues (Arg and Lys) in higher density [31]. In general, the presence of basic amino acids and a high charge at physiological pH are thought to be the most striking features of PTDs; however, there are some exceptions, suggesting that the transduction machinery may not be so simple. Various hypotheses have been proposed about what makes it possible for these peptides to translocate across biological membranes. A diverse set of experiments suggests the possibility of more than one contributing mechanism of transduction [31,33–45].

Among the TatP entry mechanisms proposed is that TatP may be able to spontaneously translocate across the cell membrane, as suggested by several experiments that indicate non-endocytosis mediated a non-energy-dependent process for their entry [33]. The TatP is a strongly charged, highly hydrophilic peptide; therefore, it is difficult to understand how this peptide can cross the hydrophobic barrier imposed by the cell membrane. It has been shown experimentally that the secondary structure of TatP does not influence its transduction capability because the *L*-and *D-*amino-acid sequence, as well as the reverse TatP sequence, can translocate through the membrane [46,47]. Substitution of any of the TatP cationic residues with neutral Ala decreases the activity, while substitution of neutral residues has no effect [47]. These observations indicate the importance of electrostatic interactions between cationic TatP and anionic phospholipid membranes [34,35,48]. The TatP is shown to interact strongly with localized negative charges in both anionic and neutral zwitterionic lipids, such as 1,2-dioleoyl-sn-glycero-3-phosphocholine and 1,2-dioleoyl-sn-glycero-3-[phospho-l-serine]; however, comparatively, a higher concentration of TatP is required for their translocation [34]. Crystallographic study showed that aqueous solutions of simple phospholipids and TatP undergo a phase transition from a lipid bilayer to a Pn3m double-diamond phase, where the lipid molecules have induced negative Gaussian curvatures and surround 6-nm water pores [34,49]. The transduction activity of TatP correlates with its ability to induce negative Gaussian curvature. Molecular dynamics simulations also suggest that strong interactions between the TatP and the phosphate groups on both sides of the lipid bilayer occur. Also present are the insertion of charged side chains that nucleate the formation of a transient pore, followed by the translocation of the Tat peptides by diffusing on the pore surface [36]. Similar to the crystallographic study in [34], a simulation study also suggests that a relatively high concentration of peptides per lipid (P/L = ∼1/8) is required for their plasma membrane translocation. Collectively, these finding challenge the hypothesis that the transduction activity of TatP can be explained solely by its ability to perform direct membrane translocation.

According to another mechanism for the TatP entry, cell surface heparin sulfate (HS) proteoglycans (HSPGs) have been shown to play a certain role in the transduction of the peptide [37–40]. HSPGs are expressed in almost all of the cell types [50]. The relationship between TatP/TatP-fusion proteins and HSPGs are shown in the following experiments: First, treatment of the cells is performed with heparinase III, which removes the HS and significantly suppresses the efficacy of TatP transduction [51]. Second, when the heparin is added, a soluble analogue of HS glycosaminoglycans (GAGs) inhibits TatP transduction [52]. *In vitro* experiments with sodium azide, an inhibitor for the production of ATP and the endocytotic pathway, have been shown to suppress the TatP/TatP-fusion protein transduction, suggesting that TatP transduction may be induced through endocytotic pathways [41,42]. Furthermore, the protein transduction was significantly inhibited by energy depletion in cells at low temperatures [43,44]. In addition, the initial study suggested that TatP enters the cells with a non-energy dependent entry; however, this result has now been shown to be an experimental artifact caused by the cell fixation process [53]. Additionally, it has been shown that the peptide is incorporated into the acidic compartment of the cells and the TatP transduction efficacy is strongly reduced by the inhibition of endosomal acidification [41].

Wadia and Dowdy invented very sophisticated systems, using a transducible TatP-Cre recombinase and a loxP-stop-loxP reporter assay on live cells [45]. These investigators showed that after an initial ionic cell-surface interaction, TatP-fusion proteins are rapidly internalized by lipid raft-dependent macropinocytosis. They reported that transduction was independent of caveolar- and clathrin-mediated endocytosis and phagocytosis. On the other hand, Richard *et al.* [44] and Fittipaldi *et al.* [54] reported clathrin- and caveolin-dependent uptake of TatP, respectively.

When we summarized the issues about the TatP cell entry mechanisms, we found that several debates have ensued for over a decade. These include (i) energy dependent vs. non-energy dependent; (ii) receptor-dependent surface binding vs. direct plasma membrane binding and translocation; and (iii) clathrin-dependent vs. caveolin-dependent endocytosis or macropinocytosis.

As mentioned above, the pathway by which these peptides enter cells is still controversial. This controversy could result from a lack of cutting edge techniques. Biological assays have shown that TatP can enter into the cells at a relatively low concentration (∼5 nM) [45]. In live cell imaging experiments, photobleaching of GFP- and FITC-labeled TatP has been used to explore the mechanisms of TatP entry. The high concentrations (∼100 nM to 10 μM) of TatP used in these experiments could induce the nonspecific entry of TatP into cells [51]; for example, pinocytosis continuously occurs at the cell surface, even without stimulation. Increasing the TatP concentration might accelerate this nonspecific entry and prevent the discovery of a truly specific entry pathway. Thus, it is difficult to identify specific entry pathways using conventional live cell imaging and photobleaching fluorophores. Moreover, the absence of any sophisticated techniques for direct visualization and the initial events at the cell surface have only been indirectly predicted based on biological activities (e.g., activity of the reporter genes) or the amount of TatP inside the cells after ∼1 h of TatP exposure. As a result, phosphate groups of plasma membrane lipids or cell-surface receptors, namely CXCR4 [55], integrin family members [54,56,57], vascular endothelial growth factor receptors [57], low density lipoprotein receptor-related proteins [58], and HSPGs [37–39,44], are still considered potential candidates. Thus, no uniform conclusions have been reached, even about the initial TatP cellular surface binding mechanisms.

## 3. Single Molecular Tracking (SMT) Methods and Usage of QD Probes

To examine the behavior of individual molecules, two methods have been applied: (i) the single-particle tracking (SPT) method, which uses a probe that is slightly larger than colloidal gold particles [59]; and (ii) single fluorescent-molecule tracking (SFMT), which uses fluorophores as probes [60,61]. By early 1990, owing to technical limitations, SPTs have been utilized for examining the behavior of individual molecules; thus, the biomolecules are attached to relatively large surface particles and observed under interference contrast microscopy. The SPT is characterized by high time-resolution. However, because relatively large particles are used as a probe, it is rather difficult to eliminate the possibility that the observed molecular behavior could be influenced by steric hindrance. SFMT was first successful in 1995 [62], with ∼20–50 nm spatial precision, but at that time, it could not detect movement of this molecular size.

Recently, the usage of QDs for visualizing single molecule behavior has dramatically changed this field [28,63]. This usage results from the invention of functional colloidal QDs, and the usage of the QD largely expands the range of single molecular observations. In addition, the development of many methods for functionalizing the surface make it possible to effectively label a wide variety of molecules with QDs [29,64]. Their moderate size (5–50 nm), higher brightness, and strong resistance to photobleaching make QDs a favorable compromise between using large beads and small yet poorly photostable organic dyes to achieve single molecular imaging analysis in living cells. Additionally, multicolor probes can be used due to the size-dependent tunable absorption and emission in the visible and near infrared (NIR) regions. As a result, utilizing QDs can open up a new gateway for “multi-parametric” single molecular imaging experiments.

**Figure 1.**
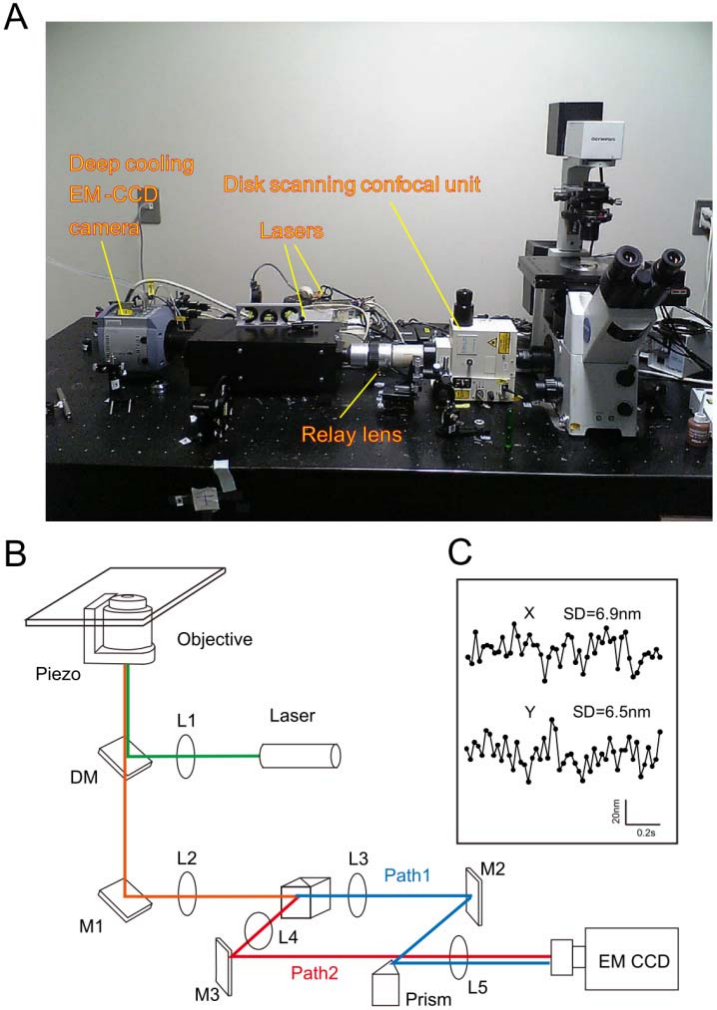
Optical system for single molecular imaging. (**A**) Photograph; (**B**) Schematic drawing of an optical setup for double imaging. A piezo actuator bound to the objective. L, lens; M, mirror; DM, dichroic mirror; HM, half mirror cube. Fluorescent images at two different wavelengths of QD (path 1 and path 2); (**C**) spatial precision of TatP-QDs in HeLa. Immobile TatP-QDs were tracked in HeLa. The S.D. of the position of QDs was 6.9 nm in the x axis and 6.5 nm in the y axis.

When a specific molecular reaction utilizing an ensemble method is analyzed, information on heterogeneous behavior, diversity in reaction, and local heterogeneity is often lost [65]. Biochemical reactions are, moreover, hardly synchronized inside of living cells; thus, it is always difficult to identify specific reaction steps from the information that is obtained by the assembling methods. The SMT is especially suitable for examining biochemical reactions that have spatial and temporal heterogeneity inside of living cells. In fact, multiple cellular events can be viewed as single molecular events because those cellular responses are triggered by a limited number of molecules. Thus, SMT is an extremely important tool to explore biological events, especially the existence of local heterogeneity, such as the molecular events of neurotransmitters inside of synaptic clefts and antigen recognition between immunological cells [28]. As compared to fluorescence recovery after photobleaching (FRAP), SMT is expected to authentically analyze molecular events under physiological conditions because the overexpression of labeled molecules is not needed. Overexpression of the molecules usually accompanies the risk of increasing the molecular density, which significantly influences the equilibrium of the biochemical reactions. Organic dyes and fluorescent proteins are typical fluorophores used to label molecules. However, despite their small size and their widespread availability in a variety of colors, organic dyes suffer from limitations such as narrow excitation band, small Stokes shift, broad fluorescence band, and photobleaching [64]. In contrast, broad absorption bands of QDs provide two advantages: (i) freedom to select any excitation wavelength below the band gap energy; and (ii) the ability to minimize the background by increasing the Stokes shift. Narrow photoluminescence bands of QDs are advantageous for minimizing bleeding during multiplexed imaging. Bright and stable photoluminescence of QDs permits durable and sensitive bioimaging even at single-molecule levels. Therefore, a much higher signal-to-noise ratio visualization can be achieved compared to organic dyes. To this end, QD-molecule labeling allows the observation of fluorescent signals for several orders of magnitude longer than fluorescent dyes and proteins, *i.e.*, between several minutes to hours. Enhancing fluorescence intensity by using QDs, together with reducing vibration and background light of microscopes and noise of cameras, can achieve higher spatial precision; therefore, the motion of the molecules can be observed over a long period of time with high positional accuracy. For example, in our laboratory, we constructed a device that reduces the vibration of the objective lens and the microscope stage, which are the main sources of vibration [32,66–68]. We used a higher quality optical filter, which transmits only the fluorescent light from the laser, to minimize the background light. Additionally, to obtain bright images with less noise, we use an Electron Multiplying CCD (EMCCD) camera with a cooling system for electronic amplification (Figure 1(A,B)). It is possible to accurately examine a molecular position by determining the light-centroid of the fluorescent spots by fitting the fluorescence intensity into the Gaussian distribution. When the number of photons (n) is 100,000, the fluorescence wavelength (λ) is 600 nm, and the numerical aperture (NA) is 1.3, the theoretical positional accuracy of the light-centroid with the Gaussian function is d = 0.88 nm (because the positional accuracy is inversely proportional to the square root of the number of photons); thus, “fluorescence imaging with one-nanometer accuracy” (FIONA) can be achieved [69,70]. In fact, because of the non-flatness of the mirror and the lens or dust, the resolution appears to have fallen to d = ∼1 nm. When we utilize very strong excitation light for visualizing a single QD, the expected photons for ∼1 nm precision can be obtained in about 5 ms of time resolution. In fact, considering the phototoxicity of the cells, we reduce the excitation light intensity to 1/100 and collect data at a video rate of (∼30 ms). We can conduct single molecule imaging of QDs with positional accuracy of ∼6 nm (Figure 1(C)) [32,67,71]. In addition, by shifting the objective lens of the confocal laser microscope up and down with a piezo controller in a multi-speed apparatus, we obtain a confocal image of nine per 330 ms, and a single three-dimensional image is created. Then, to obtain the three-dimensional position of the QDs, we have developed software that calculates the centroid of the fluorescent spot weights, to obtain their intensity and to perform a three-dimensional measurement of the position changes over time with ∼10 nm spatial precision of the Z-section [67,68].

## 4. Extracting the Meaning of Single Molecule Analysis of the Cell Membrane

To extract biological meaning from the trajectory of a molecule, a variety of analytical techniques have been developed. A commonly used method for analysis is to calculate the diffusion coefficient as a function of time, which can be used to rapidly calculate a form of motion (e.g., diffusive, direction, limitation) as well as identify additional parameters (e.g., diffusion coefficients, speed of transportation, limit sizes) [72,73]. Analysis of SMT revealed many new findings with respect to mechanisms of localization and movement of molecules, especially at the surface of the plasma membranes. However, observations of molecules inside the cells are currently limited because currently available conventional technology is unable to sufficiently incorporate fluorescent dyes and QDs inside the cells, *i.e.*, micro/nanoinjection. For visualizing intracellular molecules, there is currently enthusiasm for both specific and non-specific introduction of QDs. Simple techniques such as PTD for intracellular protein labeling with QDs will be subject to enthusiastic development in the near future.

**Figure 2.**
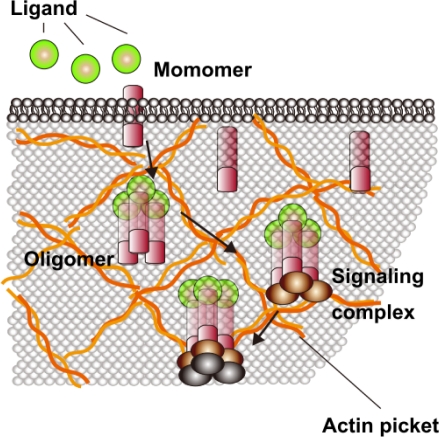
A schematic presentation of the membrane-skeleton fence model. Membrane proteins are sticking out into the cytoplasm, and in their cytoplasmic domains, collide with the membrane actin skeleton, which causes confinement of these membrane proteins into membrane skeletal meshes. When extracellular ligand molecules are added, they are bound to a receptor monomer and form oligomers. This action induces a dramatic decrease in the diffusion rates of such signaling complexes because of the enhanced effects of corralling or binding to the fences, and these signaling complexes are immediately arrested within the compartment where the signal was received.

Because the cell membrane is a liquid environment and consists of phospholipids, with proteins present in the lipid bilayer, the membrane normally conducts random diffusion. If there is no specific molecular mechanism, then anomalous diffusion, such as uneven diffusion or a stoppage of diffusion, should not occur. Using SPT, Kusumi and their colleagues found that transmembrane proteins (e.g., transferrin receptor, α2 macroglobulin receptor, cadherins, Band 3, the μ-opioid receptor, CD44) showed abnormal thermal motion in all of the examined cell lines [74–79]. The proteins showed abnormal diffusion with suppressed free movements rather than simple Brownian movements. The studies revealed the following facts: (i) the actin cytoskeleton binds the entire cytoplasmic surface of the plasma membrane and forms the membrane skeleton mesh; and (ii) membrane proteins stick out into the cytoplasm, and their cytoplasmic domains collide with the membrane skeleton, which causes confinement of these membrane proteins (Figure 2) [74,75,77,79].

To this end, a receptor molecule moves very rapidly within a compartment for a few seconds or more on average, and then hops to an adjacent compartment. Therefore, macroscopic diffusion of receptor molecules occurs as a result of a series of steps involving intercompartmental hop diffusion. This model of a “membrane skeleton fence” has now been widely accepted. Membrane skeleton-induced diffusion barriers appear to be important for maintaining spatial information for signals from the receptor molecules. For example, when an extracellular signal is bound to a receptor monomer and it forms oligomers, this formation would induce a dramatic decrease in the diffusion rates of such signaling complexes because of the enhanced effects of corralling or binding to the pickets and fences, and these signaling complexes would immediately be arrested within the compartment when it receives a signal (the oligomerization-induced trapping model) (Figure 2) [77,78,80,81]. Such an arrest may be the basis for the spatial distribution of a certain receptor after it receives a signal.

As described above, by using an SMT, it can be easier to reveal the molecular mechanisms that are linked to molecular localization and movement on the cell surface. Little by little, however, as we discover the mechanisms for cell surface molecular dynamics, it becomes possible to identify or infer the mechanisms that cause the dynamics, based on our previous findings.

## 5. Sharing the Findings of TatP Surface Tracking as an Example of Single Particle Tracking Using QDs

Developing a technology for delivering QDs into living cells is the most extensively studied research area in recent years; therefore, two different groups of researchers have already examined whether QDs conjugated with TatP can transduce QDs into living cells. Ruan *et al.* reported that TatP-QDs were first accumulated at filopodia and were eventually transported inside the cells along the microtubule tracks to the microtubule organizing center in the perinuclear region [82]. These investigators also reported that by exposing the cells to TatP-QD at 4 °C or after disrupting the cytoskeleton, the delivery of QDs was abrogated, suggesting that the conjugates were taken up by macropinocytosis. More recently, Chen *et al.* reported the intracellular delivery of QDs by TatP in human alveolar basal epithelial carcinoma cells (A549); this delivery was significantly suppressed by a 4 °C exposure, the inhibition of ATP by NaN3/2-deoxy-D-glucose, or cholesterol depletion [83]. Those studies examined whether QDs were incorporated into the cells by TatP at a specific time.

In the following section, we explain in detail our recent finding on molecular mechanisms for TatP to enter cells utilizing FIONA methods.

### 5.1. TatP Labeled with QDs Explores a New Phenomenon: Efficient Cell-Surface Binding Can only Be Achieved by Multivalent TatP

Labeling TatP with QDs, we attempted to directly visualize the initial molecular dynamics on the cell membrane. This attempt included studying the moment of TatP binding on the living cell surface, at physiological concentrations, and we attempted to clarify the molecular mechanisms that cause the protein transduction.

To visualize the interactions between TatP and the cell surface, we used TatP labeled with QDs [32]. The labeling of TatP with QDs is conducted by commercially available St-QDs coupled with biotin-TatP. As a rule, when biotin TatP is coupled with St-QD at a TatP/QD molar ratio of n:1, where n (number) of TatP is bound on the QD surface, the n-valent of TatP-QD is created. We first coupled the St-QDs with biotin-TatP at a TatP/QD molar ratio of 1:1 (designated as 1-val TatP-QDs), exposed them to the HeLa cells for 30 s, and observed them under a Nipkow-type confocal microscope at a time resolution of 400 ms/frame. The detailed results of the studies indicate that monovalent TatP has little to no capacity to bind to the surface of HeLa cells because each St-QD typically contains 5–10 streptavidin molecules and 20–40 biotin-TatP molecules that can bind to each St-QD [32]. The cells were precultured with 30 pM monovalent TatP-QDs, and different concentrations of biotin-TatP were then added to the medium. We expected that the extra biotin-TatP in the medium would bind to the free streptavidin sites on the TatP-QDs. As expected, the binding of TatP-QD on the cells is induced, and the numbers of TatP-QDs that are bound to the cell surface increased proportionately along with the concentrations of biotin-TatP in the medium, up to 5 μM (this concentration is the highest that we tested so far). In contrast, the addition of unlabeled TatP to the medium at concentrations of up to 50 μM did not significantly enhance the ability of TatP-QDs to bind to the cell surface. These results suggest that the number of TatPs on the QDs could determine the capability for cell-surface binding. Moreover, the finding that increasing the non-biotin TatP could not increase the binding capability of monovalent TatP-QD indicated that direct interactions between TatP in the medium and TatP on QDs could not be induced (or very little); thus, the valence of TatP on the QD surface did not change. These results also support the earlier findings that TatP does not form an oligomer [42].

**Figure 3.**
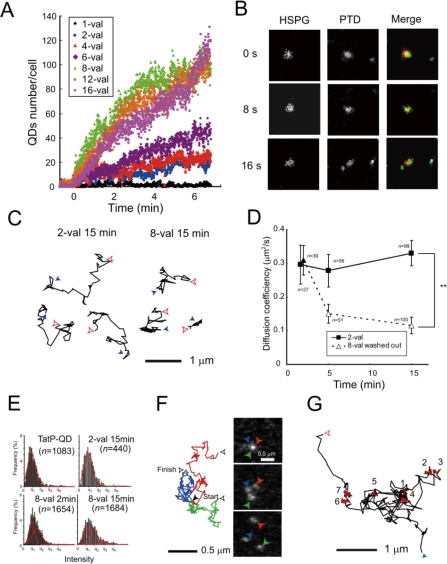
Single TatP-QD tracking on HeLa cells. (**A**) Typical time course of cell binding for TatP-QD, with different valences, to HeLa cells. The cells were exposed to TatP-QDs of the indicated valence or medium, and the images were collected at 400 ms/frame; (**B**) Time course fluorescence images obtained using a dual imaging system at 200 ms/frame. Fluorescence images of TatP-QD525 (lower left panel) and HSPG as detected by F58-10E4 plus secondary Ab labeled with QD705 (lower right panel) at 20 min of TatP exposure; (**C**) Typical trajectories of 2- or 8-val TatP after 15 min of exposure, recorded at 23 ms/frame; (**D**) Mean diffusion coefficient of 2- or 8-val TatP, over time up to 15 min, averaged for all particles in time window 1150 ms; (**E**) Intensity histogram of TatP-QDs at the indicated time and condition, fitted to four Gaussian curves. The mean of the first peak is equal to TatP-QD (left upper panel), defined as q1; (**F**) Selected frames (left) and trajectories of 8-val TatP-QDs from 23 ms/frame after a 15-min exposure. Arrowhead color corresponds to that of its trajectory; (**G**) 8-val TatP often exhibited alternating periods of apparently simple Brownian diffusion (black) and temporary immobilization (red). ** *P* < 1 × 10^−7^ Reproduced with permission from reference 32. Permission is required from copyright owner.

To test whether the increase in TatP peptides per QD enhances cellular binding, we examined the cell surface association of QDs with different valences of TatP utilizing single particle observations (Figure 3(A)). These results demonstrate that increasing the TatP valency on the QDs enhances cellular binding. We found that at least bivalent TatP-QD is required to support TatP-QD cell-surface binding on living cells at a minimum of 30 pM in concentration, and a valence of 8 is required for rapid and efficient cell-surface binding of TatP. Ryser *et al.* also obtained similar findings: they first demonstrated an enhancement of cellular uptake for cationic peptide polymers by multivalency [84]. More recently, they found that the level of conjugation of HIV-1 Tat peptides to superparamagnetic beads influences their cellular uptake in lymphocytes [85]. Thus, utilizing the single particle visualization technique, we are able to directly analyze TatP binding on the living cell surface at as low as 30 pM concentration, thus avoiding nonspecific uptake into cells. Moreover, by measuring the number of TatP-QD particles per second from the visualization data recorded at 400 ms/frame, we can easily measure the binding velocity of different valences on living cells.

In general, multivalent interactions are characterized by the simultaneous binding of multiple ligands on one biological component (such as a molecule and a surface) to multiple receptors on another. These types of interactions occur throughout biological systems and have a number of characteristics that monovalent interactions do not have. Specifically, multivalent interactions can be collectively much stronger than the corresponding monovalent interactions; however, molecular mechanisms on the multivalent interactions and their biological effects are not yet fully understood. Utilizing single particle observations of events on plasma membranes, we can now quantitatively analyze the relationship between the binding velocity and multivalence in living cells. At the same time, we can also analyze the involvements of various cellular mechanisms by dissecting different steps of the TatP entry into living cells. Collectively, these results suggest the possibility that further improvements to single molecule microscopy might enable the measurement of the molecular binding constant on the living cell surface. In other words, simultaneous measurement of both the physical and the biological effects of multivalent interactions by single molecule microscopy may reveal the entire picture of the molecular mechanisms.

### 5.2. Dual Imaging of TatP Labeled with Two Different-Wavelength QDs Confirms that HSPGs Are Essential Receptors for TatP Cell-Surface Binding

Does TatP enter a cell through receptor-dependent or receptor-independent direct lipid bilayer translocation? Several recent studies have shown that cell-surface HSPGs are involved in Tat transduction into cells. HSPGs are composed of a core protein and ≥3 linear HS-GAG polysaccharide chains [50]. To answer this question, we simultaneously observed two single particles of TatP, TatP-QD525 and HSPGs labeled with QD705-labeled anti-HSPGs, in living cells using single molecule microscopy with dual imaging techniques.

Before proceeding to dual imaging, we first studied the effect of heparin, with highly sulfated GAG, on the cellular binding of TatP. Single particle observation revealed that the cell-surface binding of 8-val TatP was totally abrogated by the presence of heparin in the medium. To clarify the involvement of HSPGs in the cell-surface binding of TatP, we examined the effect of removing cell-surface GAGs with specific enzymes. We found that the treatment with HS lyase, which cleaves GAG from HSPG, totally abrogates 8-val TatP cell-surface binding to HeLa cells. Collectively, these results suggest that the GAGs of HSPGs serve as the initial receptors for TatP on the cell surface. Then, finally, confirming the role of HSPGs as receptors for TatP with the dual imaging techniques was required. After exposure, 8-val TatP-QD525 co-mobilized with HSPGs labeled with QD705-labeled anti-HSPGs antibodies at the cellular surface, and TatP-HSPGs were subsequently co-mobilized inside the cells within 45 min of 8-val TatP exposure (Figure 3(B)). Combined with the result of enzymatic removal of HSPGs, the dual imaging of these two single particles confirms that the cell-surface HSPGs serve as the initial TatP receptors, and that the TatP-HSPGs complex transports TatP inside the living cells. Thus, using single-molecule visualization, we finally conclude the long-debated question of the TatP initial cell surface binding mechanisms. Additionally, in agreement of the observation by Ruan *et al.* that TatP-QDs were initially accumulated at filopodia [82], we also found that HSPGs were abundant on the filopodia and lamellipodia.

### 5.3. Signaling from Multivalent TatP Facilitates the Frequent and Constitutive Recruitment of HSPGs to Actin-Associated Membrane Lipid Rafts

The intensity distribution, trajectory, proportion of mobility, and the mean diffusion calculated from each TatP leveled QD are the key aspects of the single molecule experiment. Many unresolved problems can be explored by simple trajectory analysis of each QD.

Quantitative thermodynamic description by isothermal titration calorimetry has revealed that TatP has a binding affinity for HS and induces crosslinking and/or aggregation of HS-TatP complexes in liquid medium [40]. Thus, it was reasonable for us to hypothesize that multivalent TatP induces crosslinking of HSPGs on the cell surface immediately after binding. To characterize the molecular behavior of TatP/HSPGs in the absence of crosslinking, we observed the movement of both 2-val TatPs, which cannot induce the crosslinking of receptors, and 8-val TatP, which can crosslink the HSPGs with ∼7 nm spatial precision. The trajectory, the proportion of mobility, and the mean diffusion values for 2- and 8-val Tat were compared. The intensity distributions of the visualized spots were fitted to multiple Gaussian curves and were compared with the distributions of a single QD. We then found that cell-bound 2-val TatP consists of single QD molecules after an exposure of 2 and 15 min. In contrast, the trajectory, the proportion of mobility, and the mean diffusion of 8-val TatP-QDs were almost identical to those of 2-val TatP particles until 5 min. However, these parameters were found to drop substantially over time until 15 min, indicating that the compartmentalization of the mobility of TatP-QDs occurs on the cell surface (Figure 3(C,D)). The intensity distributions of the cell-bound 8-val TatP particles were found to be gradually increased, suggesting that they consisted of more than two QDs (designated here as spots_>2_ QDs) (Figure 3(E,F)). We found that, after partial actin depolymerization (by latrunculin-B) or partial cholesterol depletion (by methyl-β-cyclodextrin, MBC), the reduction of the diffusion of the 8-val TatP and the appearance of the spots_>2_, the QDs almost completely disappeared. Collectively, these results indicate that the reduction in the movement of the 8-val TatP/HSPGs and the increase in the number of spots_>2_ QDs were linked to the actin filaments and the cholesterol-rich domains in the membrane, known as lipid rafts or membrane microdomains.

When we analyzed the mobility of 2-val and 8-val TatP-QD for longer periods with 23 ms/frame, after 15 min of exposure, we found that only 8-val TatP particles often exhibited very abnormal movements, namely, 8-val TatP undergoes alternating periods of simple Brownian diffusion and temporary immobilization in its trajectories (Figure 3(G)). These temporary pauses persisted for nearly half of the total observation time, and thus, the temporary immobilization-to-diffusion ratio was 1:2. This mobility is, surprisingly, almost identical to that of colloidal gold-induced crosslinking of CD59, and the temporary immobilization is designated as a stimulation-induced temporary arrest of lateral diffusion (STALL) [80,81]. By performing detailed experiments, Suzuki *et al.* revealed the molecular mechanisms for STALL in the crosslinking of CD59. The crosslinking of CD59 exhibited a frequent but transient recruitment in the lipid raft domain via both protein-protein and lipid-lipid (raft) contributions, and CD59 underwent alternating periods of actin-dependent STALL. At the lipid raft, the signaling molecules also formed into CD59 clusters and appeared to activate an unknown protein thereafter. CD59 clusters are indirectly associated with the actin cytoskeleton which has lipid membrane microdomains via protein-protein interactions; these are likely to have a key function in converting extracellular CD59 signals to the intracellular Ca^2+^ signaling pathways. When applying this finding to our results, 8-val TatP-QD/HSPGs were frequently recruited to lipid microdomains and activated intracellular signaling molecules using some unknown mechanisms, which might cause frequent but transient recruitment to the actin cytoskeleton with lipid membrane microdomains. As a result, 8-val TatP-QD/HSPGs are frequently recruited and are stopped at the lipid raft, and thus appear to link to induce spots_>2_ QDs. Indeed, the STALL-like mobility of 8-val TatP was also inhibited by both partial depolymerization of the actin filaments and partial cholesterol depletion, indicating that this action was induced by mechanisms similar to STALL.

### 5.4. Multivalent TatP Activates Rac1 to Slow Molecular Movement and Induce Internalization through Macropinocytosis

Full-length Tat protein exposure activates Rac1, a GTP-binding protein that regulates membrane actin polymerization [57]. We hypothesized that the crosslinking of HSPGs by 8-val TatP causes the recruitment and activation of Rac1 adjacent to the lipid rafts and the membrane actin near the raft domains, and regulates membrane actin polymerization, resulting in reducing the diffusion of TatP-QD/HSPG and also activating some mechanisms for taking up TatP-QD/HSPG from the cell surface.

**Figure 4.**
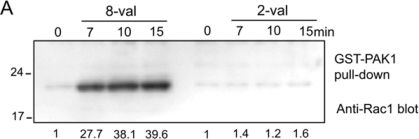

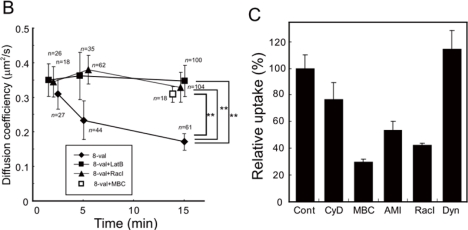
Activation of Rac1 by 8-val-TatP exposure linked to slowerig diffusion and TatP internalization by macropinocytosis. (**A**) levels of activated Rac1 after 8-val or 2-val TatP-QD exposure. HeLa cells, Lysates were harvested, and Pak1 pulldown was performed. Samples from pulldowns were loaded and blotted with anti-Rac1. The numbers indicate the level of Rac1 activation; (**B**) mean diffusion coefficient of 8-val TatP in the presence and absence of LatB, Rac1 inhibitor (RacI), and MBC. Bars, S.E. ** p < 1 × 10^−15^ as calculated by Student’s t test; (**C**) effects of cytochalasin D (CyD), MBC, amiloride (AMI), Rac1 inhibitor (RacI), and Dynasore (Dyn). Bars, S.D.

We first measured the amplitude and the time course of Rac1 activation in the cells exposed to 2-val and 8-val TatP and found that 8-val TatP-QDs activates Rac1 within 7 min of exposure, continuing until 15 min of exposure. In contrast, the induction of Rac1 activation was marginal in cells that were exposed to 2-val TatP (Figure 4(A)). Furthermore, Rac1 was found to be clustered with TatP-coated beads and not with uncoated beads, confirming that the crosslinking of HSPGs by 8-val TatP recruited Rac1 to the membrane. We also studied the mobility of individual 8-val QDs in cells that were pre-exposed to NCS23766, a Rac1 inhibitor (Figure 4(B)). We found that the reductions of average diffusion and the induction of STALL-like events were suppressed by the Rac1 inhibitor. The gradual increase in the number of the spots_>2_ QDs over time was also found to be significantly suppressed by the inhibitor.

These results indicate that there was crosslinkage of the HSPGs by TatP, activating Rac1 and causing membrane actin polymerization. We then examined whether Rac1 activation by multivalent TatP is linked to the internalization of TatP/HSPGs. We found that the internalization of 8-val TatP is suppressed by inhibiting actin polymerization (cytochalasin D) and Na+/H+ exchange, which are required for macropinocytosis (amiloride), and is also suppressed by changing cholesterol levels with MBC, as reported previously. The inhibitor for dynamin (Dynasore), a GTPase that is responsible for most of the endocytotic pathways, had no effect (Figure 4(C)). From these data, we can confidently assume that the exposure of cells to 8-val TatP induces the internalization of TatP through macropinocytosis.

**Figure 5.**
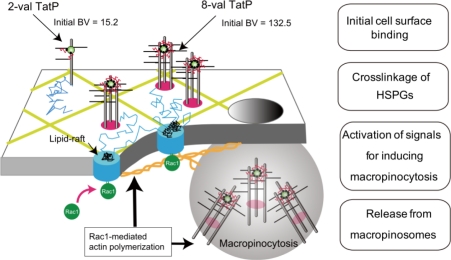
A schematic presentation of the events in the plasma membrane exposed to 2-val and 8-val TatP. HSPG exists as a monomer; 8-val TatP clusters with several HSPGs, leading to recruitment of Rac1 to lipid rafts, which triggers membrane reorganization of actin filaments and/or actin-dependent domains and induces the temporary immobilization and clustering of 8-val TatP/HSPGs. Rac1 activation induces TatP/HSPG internalization through macropinocytosis. The major sequential rate-limiting steps of TatP intracellular delivery are also described. Numbers of the initial binding velocity (BV) shown here are in arbitrary units. The right column indicates the representative rate-limiting factors.

By the introduction of single-QD tracking with FIONA, we can now directly observe the molecular events of TatP in physiological concentrations; therefore, we are able to directly prove that “TatP induces the signals and changing molecular motion of the TatP/HSPGs and is incorporated into cells through macropinocytosis”. These visualization techniques can also be used to directly prove the location of the rate-limiting step for protein transduction. Therefore, the direct identification of a rate-limiting step of the PTD entry now can be possible, which is crucial to improving the efficiency of PTD based on knowledge gained from the experiments, rather than relying on observations of the biological output (Figure 5). Thus, single QD visualization of PTDs will greatly contribute to improving observations on their transduction efficacy. Moreover, this technology is expectedly to contribute to the improvement of intracellular labeling technology with QD.

## 6. Problems with QD and Challenges for the Future

The superior photoluminescence properties of QDs make it possible to observe the mobility of TatP at the molecular level, at physiological concentrations. Despite these superior characteristics, it was also revealed that there are several serious drawbacks in the use of QDs. We will discuss here briefly the photoluminescent problems, the functionalization and hydrodynamic size of the QD, and possible methods for the solution to these problems as well.

### 6.1. Blinking of QDs

Blinking of QDs is a phenomenon in which photoluminescence turns “on” and “off” intermittently, even under continuous photoexcitation [28,61,86]. This phenomenon is an intrinsic limitation to the advancement of QD technology toward single-molecule biophysical and biochemical investigations. Blinking is quite troublesome, especially for tracking a molecule for longer periods because trajectories of labeled molecules must be reconstructed from a transiently disappearing signal. Thus, it is almost impossible to track down a single QD for longer periods regardless of its extremely photoresistant nature. It is important to understand the underlying fundamental mechanisms for blinking, and they are currently being extensively investigated at both the theoretical and practical levels [87–90]. Although the details are still unclear, a consensus has emerged that photoluminescence blinking is fundamentally caused by intermittent Auger ionization, which results in the formation of positively charged QDs. Because of strong Coulombic interactions, successive photoactivation of a charged QD results in nonradiative carrier recombination, inducing long-lived ‘off’ states in the photoluminescence intensity. Various approaches have been investigated to address and suppress the blinking photoluminescence of QDs, including the effects of novel metal nanoparticles/surfaces, temperature, a shell thickness of higher band gap materials, electron transfer to and from inorganic NPs and organic molecules, capping by thiols and polymers, oxygen depletion, modified synthesis, and excitation energy and intensity [29]. Among the relevant efforts, Wang *et al.* recently accomplished the synthesis of a completely nonblinking ternary core/shell CdZnSe/ZnSe QD by circularly alloying CdZnSe into ZnSe [91]. It is extremely important to invent innovative methods for suppressing the blinking of single QDs. However, researchers have now proceeded despite this difficulty, for the sake of isolating meaningful information from the currently available QDs, which undergo blinking. They attempt to overcome blinking with the serial tracking of QDs until they blink off; in other words, they are observed for a short period at different time intervals and then are reconstituted into a continuous series of single TatP-QD mobility measurements from these data. Another option for overcoming the blinking issue is utilizing aggregated QDs [67]. If QDs aggregate, the mean off-state time is shortened because of overlapping blinking particles. Whenever one QD is in the on-state, the entire aggregate will appear to be “on”. It might not be worth the effort to explore the influence of the hydrodynamic effects on aggregated QDs in order to understand the dynamics of the labeled molecules. Nevertheless, the tracking of human epidermal factor 2 receptors inside cells labeled with the aggregated QDs can readily identify the QD transportation by myosin VI, consisting of 29- and 15-nm steps, indicating that successful observation of myosin VI hand-over-hand movement is achieved by this method [67,71]. If the tracking down of single molecules that are labeled with nonblinking QDs can be possible in the future, then it would not be surprising to identify a novel behavior of the molecules that cannot be expected from the reconstructed data of shorter periods. Similarly, tracking of the intracellular TatP behavior for longer periods with nonblinking QDs is a potential application for identification of rate-limiting steps of PTD to enhance their transduction efficacy.

### 6.2. Functionalization and the Hydrodynamic Size of QDs

Synthesized high-quality core and core/shell QDs are usually covered by a layer of hydrophobic molecules [29,64,86]. Thus, conversion of QDs from an organic to an aqueous phase has become necessary for biocompatibility. For biological applications of QDs, the surface cap should have intact or improved optical properties, mono-dispersion in aqueous phase, functional groups for bioconjugation, and protection against chemical/biochemical/physical damage and dissolution of toxic materials. In this regard, sequential introduction of a primary shell from a higher band-gap semiconductor, a silica shell and/or a polymer shell, a PEG layer, and functional groups for bioconjugate reactions are preferable. However, the hydrodynamic size of the QDs should be controlled for a better compromise among the sizes of the QD, the binding/labeling efficiency, and the functioning of targets after labeling. Functionalization is now typically achieved by conjugating reactive biomolecules (e.g., enzymes, antibodies, nucleic acids) and polyethylene glycol (PEG), which is often necessary to minimize nonspecific interactions of the QD with biological material. Nonspecific binding is hostile for the high-contrast imaging of cells, as well as for the fateful observation of the molecular mobility of the labeled molecules. Owing to the functionalization of the surface with PEG, commercially available QDs show almost negligible levels of nonspecific binding. However, their hydrodynamic size is ∼20–40 nm, which is larger than the optimal size for tracking labeling molecules on the cellular surface. For example, when we track down the TatP-QD molecules on the ventral side, the QD could not readily enter the space in-between the ventral membrane and the cover slip, and eventually became stuck at the marginal area of the ventral surface. Thus, a much smaller size that minimizes nonspecific interactions of QDs should be invented. For this endeavor, researchers are now focused on inventing various methods for functionalizing QDs as well as synthesizing new core/shell materials for QDs.

## 7. Conclusions

By tracking, observing, and measuring the motility of the single TatP-QD using FIONA methods, we can identify critical limiting steps for TatP intracellular delivery. This method also provides information on the cell surface binding velocity of TatP-HSPGs on a living cell surface. Most importantly, these simple techniques can easily provide direct information regarding TatP cell surface binding mechanisms as well as their entry mechanisms, which have been debated for more than a decade. Moreover, our method here for direct identification of rate-limiting steps of PTD entry with SQT should dramatically improve methods for enhancing the transduction efficiency. Although more effort is needed to synthesize idealistically functionalized QDs without blinking, FIONA with labeled QDs should prove to be a valuable tool to interpret the rules governing the dynamic architecture of living cells, and will possibly reshape some important conceptual ideas in cell biology.

## Figures and Tables

**Table 1.** Amino acid sequence of protein transduction domains.

*Name*	*Species*	*Sequence*

Tat (49–57)	HIV-1	GRKKRRQRRRPPQ
Antennapedia	Drosophila	RQIKIWFQNRRMKWKK
VP22	HSV-1	DAATATRGRSAASRPTERPRAPARSASRPRRPVD

## References

[1] Frankel A.D., Pabo C.O. (1988). Cellular uptake of the TAT protein from human immunodeficiency virus. Cell.

[2] Green M., Loewenstein P.M. (1988). Autonomous functional domains of chemically synthesized Human Immunodeficiency Virus TAT trans-activator protein. Cell.

[3] Perez F., Joliot A., Blochgallego E., Zahraoui A., Triller A., Prochiantz A. (1992). Antennapedia homeobox as a signal for the cellular internalization and nuclear addressing of a small exogenous peptide. J. Cell Sci.

[4] Elliott G., Ohare P. (1997). Intercellular trafficking and protein delivery by a herpesvirus structural protein. Cell.

[5] Nagahara H., Vocero-Akbani A.M., Snyder E.L., Ho A., Latham D.G., Lissy N.A., Becker-Hapak M., Ezhevsky S.A., Dowdy S.F. (1998). Transduction of full-length TAT fusion proteins into mammalian cells: TAT-p27(Kip1) induces cell migration. Nat. Med.

[6] Fawell S., Seery J., Daikh Y., Moore C., Chen L.L., Pepinsky B., Barsoum J. (1994). TAT-mediated delivery of heterologous proteins into cells. Proc. Natl. Acad. Sci. USA.

[7] Siomi H., Shida H., Maki M., Hatanaka M. (1990). Effects of a Highly basic region of human-immunodeficiency-virus tat protein on nucleolar localization. J. Virol.

[8] Ignatovich I.A., Dizhe E.B., Pavlotskaya A.V., Akifiev B.N., Burov S.V., Orlov S.V., Perevozchikov A.P. (2003). Complexes of plasmid DNA with basic domain 47–57 of the HIV-1 Tat protein are transferred to mammalian cells by endocytosis-mediated pathways. J. Biol. Chem.

[9] Kaplan I.M., Wadia J.S., Dowdy S.F. (2005). Cationic TAT peptide transduction domain enters cells by macropinocytosis. J. Control. Release.

[10] Vives E., Brodin P., Lebleu B. (1997). A truncated HIV-1 Tat protein basic domain rapidly translocates through the plasma membrane and accumulates in the cell nucleus. J. Biol. Chem.

[11] Cao G.D., Pei W., Ge H.L., Liang Q.H., Luo Y.M., Sharp F.R., Lu A.G., Ran R.Q., Graham S.H., Chen J. (2002). In vivo delivery of a Bcl-xL fusion protein containing the TAT protein transduction domain protects against ischemic brain injury and neuronal apoptosis. J. Neurosci.

[12] Schwarze S.R., Ho A., Vocero-Akbani A., Dowdy S.F. (1999). In vivo protein transduction: Delivery of a biologically active protein into the mouse. Science.

[13] Lindsay M.A. (2002). Peptide-mediated cell delivery: Application in protein target validation. Curr. Opin. Pharmacol.

[14] Diem R., Taheri N., Dietz G.P.H., Kuhnert A., Maier K., Sattler M.B., Gadjanski L., Merkler D., Bahr M. (2005). HIV-tat-mediated BCl-X-L delivery protects retinal ganglion cells during experimental autoimmune optic neuritis. Neurobiol. Dis.

[15] Soane L., Fiskum G. (2005). TAT-mediated endocytotic delivery of the loop deletion Bcl-2 protein protects neurons against cell death. J. Neurochem.

[16] Astriab-Fisher A., Sergueev D.S., Fisher M., Shaw B.R., Juliano R.L. (2000). Antisense inhibition of P-glycoprotein expression using peptide-oligonucleotide conjugates. Biochem. Pharmacol.

[17] Sakaguchi M., Nukui T., Sonegawa H., Murata H., Futami J., Yamada H., Huh N. (2005). Targeted disruption of transcriptional regulatory function of p53 by a novel efficient method for introducing a decoy oligonucleotide into nuclei. Nucleic Acids Res.

[18] Futaki S., Ohashi W., Suzuki T., Niwa M., Tanaka S., Ueda K., Harashima H., Sugiura Y. (2001). Stearylated arginine-rich peptides: A new class of transfection systems. Bioconjug. Chem.

[19] Ostensen C.G., Sandberg-Nordqvist A.C., Chen J., Hallbrink M., Rotin D., Langel U., Efendic S. (2002). Overexpression of protein-tyrosine phosphatase PTP sigma is linked to impaired glucose-induced insulin secretion in hereditary diabetic Goto-Kakizaki rats. Biochem. Biophys. Res. Commun.

[20] Pooga M., Soomets U., Hallbrink M., Valkna A., Saar K., Rezaei K., Kahl U., Hao J.X., Xu X.J., Wiesenfeld-Hallin Z. (1998). Cell penetrating PNA constructs regulate galanin receptor levels and modify pain transmission in vivo. Nat. Biotechnol.

[21] Simeoni F., Morris M.C., Heitz F., Divita G. (2003). Insight into the mechanism of the peptide-based gene delivery system MPG: Implications for delivery of siRNA into mammalian cells. Nucleic Acids Res.

[22] Polyakov V., Sharma V., Dahlheimer J.L., Pica C.M., Luker G.D., Piwnica-Worms D. (2000). Novel Tat-peptide chelates for direct transduction of technetium-99m and rhenium into human cells for imaging and radiotherapy. Bioconjug. Chem.

[23] Lewin M., Carlesso N., Tung C.H., Tang X.W., Cory D., Scadden D.T., Weissleder R. (2000). Tat peptide-derivatized magnetic nanoparticles allow in vivo tracking and recovery of progenitor cells. Nat. Biotechnol.

[24] Torchilin V.P., Rammohan R., Weissig V., Levchenko T.S. (2001). TAT peptide on the surface of liposomes affords their efficient intracellular delivery even at low temperature and in the presence of metabolic inhibitors. Proc. Natl. Acad. Sci. USA.

[25] Beeser A., Chernoff J. (2005). Production and use of a cell permeable inhibitor of group A Paks (TAT-PID) to analyze signal transduction. Methods.

[26] Fujihara S., Jaffray E., Farrow S.N., Rossi A.G., Haslett C., Hay R.T. (2005). Inhibition of NF-kappa B by a cell permeable form of I kappa B alpha induces apoptosis in eosinophils. Biochem. Biophys. Res. Commun.

[27] Heng B.C., Cao T., Tong G.Q., Ng S.C. (2004). Potential utility of cell-permeable transcription factors to direct stem cell differentiation. Stem Cells Dev.

[28] Pinaud F., Clarke S., Sittner A., Dahan M. (2010). Probing cellular events, one quantum dot at a time. Nat. Methods.

[29] Biju V., Itoh T., Ishikawa M. (2010). Delivering quantum dots to cells: Bioconjugated quantum dots for targeted and nonspecific extracellular and intracellular imaging. Chem. Soc. Rev.

[30] Noguchi H., Matsushita M., Kobayashi N., Levy M.F., Matsumoto S. (2010). Recent advances in protein transduction technology. Cell Transpl.

[31] Chauhan A., Tikoo A., Kapur A.K., Singh M. (2007). The taming of the cell penetrating domain of the HIV Tat: Myths and realities. J. Control. Release.

[32] Imamura J., Suzuki Y., Gonda K., Roy C.N., Gatanaga H., Ohuchi N., Higuchi H. (2011). Single particle tracking confirms that multivalent Tat protein transduction domain-induced heparan sulfate proteoglycan cross-linkage activates rac1 for internalization. J. Biol. Chem.

[33] Lundberg P., Langel U. (2003). A brief introduction to cell-penetrating peptides. J. Mol. Recognit.

[34] Mishra A., Gordon V.D., Yang L.H., Coridan R., Wong G.C.L. (2008). HIV TAT forms pores in membranes by inducing saddle-splay curvature: Potential role of bidentate hydrogen bonding. Angew. Chem.-Int. Ed.

[35] Tiriveedhi V., Butko P. (2007). A fluorescence spectroscopy study on the interactions of the TAT-PTD peptide with model lipid membranes. Biochemistry.

[36] Herce H.D., Garcia A.E. (2007). Molecular dynamics simulations suggest a mechanism for translocation of the HIV-1 TAT peptide across lipid membranes. Proc. Natl. Acad. Sci. USA.

[37] Rusnati M., Coltrini D., Oreste P., Zoppetti G., Albini A., Noonan D., diFagagna F.D., Giacca M., Presta M. (1997). Interaction of HIV-1 Tat protein with heparin—Role of the backbone structure, sulfation, and size. J. Biol. Chem.

[38] Sandgren S., Cheng F., Belting M. (2002). Nuclear targeting of macromolecular polyanions by an HIV-Tat derived peptide - Role for cell-surface proteoglycans. J. Biol. Chem.

[39] Tyagi M., Rusnati M., Presta M., Giacca M. (2001). Internalization of HIV-1 Tat requires cell surface heparan sulfate proteoglycans. J. Biol. Chem.

[40] Ziegler A., Seelig J. (2004). Interaction of the protein transduction domain of HIV-1 TAT with heparan sulfate: Binding mechanism and thermodynamic parameters. Biophys. J.

[41] Drin G., Cottin S., Blanc E., Rees A.R., Temsamani J. (2003). Studies on the internalization mechanism of cationic cell-penetrating peptides. J. Biol. Chem.

[42] Thoren P.E.G., Persson D., Isakson P., Goksor M., Onfelt A., Norden B. (2003). Uptake of analogs of penetratin, Tat(48–60) and oligoarginine in live cells. Biochem. Biophys. Res. Commun.

[43] Mann D.A., Frankel A.D. (1991). Endocytosis and targeting of exogenous HIV-1 TAT protein. EMBO J.

[44] Richard J.P., Melikov K., Brooks H., Prevot P., Lebleu B., Chernomordik L.V. (2005). Cellular uptake of unconjugated TAT peptide involves clathrin-dependent endocytosis and heparan sulfate receptors. J. Biol. Chem.

[45] Wadia J.S., Stan R.V., Dowdy S.F. (2004). Transducible TAT-HA fusogenic peptide enhances escape of TAT-fusion proteins after lipid raft macropinocytosis. Nat. Med.

[46] Futaki S., Suzuki T., Ohashi W., Yagami T., Tanaka S., Ueda K., Sugiura Y. (2001). Arginine-rich peptides - An abundant source of membrane-permeable peptides having potential as carriers for intracellular protein delivery. J. Biol. Chem.

[47] Wender P.A., Mitchell D.J., Pattabiraman K., Pelkey E.T., Steinman L., Rothbard J.B. (2000). The design, synthesis, and evaluation of molecules that enable or enhance cellular uptake: Peptoid molecular transporters. Proc. Natl. Acad. Sci. USA.

[48] Ziegler A., Blatter X.L., Seelig A., Seelig J. (2003). Protein transduction domains of HIV-1 and SIV TAT interact with charged lipid vesicles. Binding mechanism and thermodynamic analysis. Biochemistry.

[49] Schmidt N., Mishra A., Lai G.H., Wong G.C.L. (2010). Arginine-rich cell-penetrating peptides. FEBS Lett.

[50] Esko J.D., Lindahl U. (2001). Molecular diversity of heparan sulfate. J. Clin. Investig.

[51] Ziegler A., Nervi P., Durrenberger M., Seelig J. (2005). The cationic cell-penetrating peptide Cpp(TAT) derived from the HIV-1 protein TAT is rapidly transported into living fibroblasts: Optical, biophysical, and metabolic evidence. Biochemistry.

[52] Hakansson S., Jacobs A., Caffrey M. (2001). Heparin binding by the HIV-1 tat protein transduction domain. Protein Sci.

[53] Richard J.P., Melikov K., Vives E., Ramos C., Verbeure B., Gait M.J., Chernomordik L.V., Lebleu B. (2003). Cell-penetrating peptides - A reevaluation of the mechanism of cellular uptake. J. Biol. Chem.

[54] Fittipaldi A., Giacca M. (2005). Transcellular protein transduction using the Tat protein of HIV-1. Adv. Drug Delivery Rev.

[55] Xiao H., Neuveut C., Tiffany H.L., Benkirane M., Rich E.A., Murphy P.M., Jeang K.T. (2000). Selective CXCR4 antagonism by Tat: Implications for in vivo expansion of coreceptor use by HIV-1. Proc. Natl. Acad. Sci. USA.

[56] Barillari G., Gendelman R., Gallo R.C., Ensoli B. (1993). The TAT protein of Human-Immunodeficiency-Virus Type-1, A growth-factor for AIDS Kaposi-sarcoma and cytokine-activated vascular cells, induces adhesion of the same cell-types by using integrin receptors recognizing the RGD amino-acid-sequence. Proc. Natl. Acad. Sci. USA.

[57] Toschi E., Bacigalupo I., Strippoli R., Chiozzini C., Cereseto A., Falchi M., Nappi F., Sgadari C., Barillari G., Mainiero F., Ensoli B. (2006). HIV-1 Tat regulates endothelial cell cycle progression via activation of the Ras/ERK MAPK signaling pathway. Mol. Biol. Cell.

[58] Albini A., Soldi R., Giunciuglio D., Giraudo E., Benelli R., Primo L., Noonan D., Salio M., Camussi G., Rockl W., Bussolino F. (1996). The angiogenesis induced by HIV-1 Tat protein is mediated by the Flk-1/KDR receptor on vascular endothelial cells. Nat. Med.

[59] Saxton M.J., Jacobson K. (1997). Single-particle tracking: Applications to membrane dynamics. Annu. Rev. Biophys. Biomol. Struct.

[60] Joo C., Balci H., Ishitsuka Y., Buranachai C., Ha T. (2008). Advances in single-molecule fluorescence methods for molecular biology. Annu. Rev. Biochem.

[61] Moerner W.E., Orrit M. (1999). Illuminating single molecules in condensed matter. Science.

[62] Funatsu T., Harada Y., Tokunaga M., Saito K., Yanagida T. (1995). Imaging of single fluorescent molecules and individual atp turnovers by single myosin molecules in aqueous-solution. Nature.

[63] Bruchez M., Moronne M., Gin P., Weiss S., Alivisatos A.P. (1998). Semiconductor nanocrystals as fluorescent biological labels. Science.

[64] Medintz I.L., Uyeda H.T., Goldman E.R., Mattoussi H. (2005). Quantum dot bioconjugates for imaging, labelling and sensing. Nat. Mater.

[65] Weiss S. (1999). Fluorescence spectroscopy of single biomolecules. Science.

[66] Toba S., Watanabe T.M., Yamaguchi-Okimoto L., Toyoshima Y.Y., Higuchi H. (2006). Overlapping hand-over-hand mechanism of single molecular motility of cytoplasmic dynein. Proc. Natl. Acad. Sci. USA.

[67] Watanabe T.M., Higuchi H. (2007). Stepwise movements in vesicle transport of HER2 by motor proteins in living cells. Biophys. J.

[68] Watanabe T.M., Sato T., Gonda K., Higuchi H. (2007). Three-dimensional nanometry of vesicle transport in living cells using dual-focus imaging optics. Biochem. Biophys. Res. Commun.

[69] Yildiz A., Forkey J.N., McKinney S.A., Ha T., Goldman Y.E., Selvin P.R. (2003). Myosin V walks hand-over-hand: Single fluorophore imaging with 1.5-nm localization. Science.

[70] Kural C., Kim H., Syed S., Goshima G., Gelfand V.I., Selvin P.R. (2005). Kinesin and dynein move a peroxisome in vivo: A tug-of-war or coordinated movement?. Science.

[71] Ybo J., Kambara T., Gonda K., Higuchi H. (2008). Intracellular imaging of targeted proteins labeled with quantum dots. Exp. Cell Res.

[72] Wieser S., Schutz G.J. (2008). Tracking single molecules in the live cell plasma membrane-Do’s and Don’t’s. Methods.

[73] Qian H., Sheetz M.P., Elson E.L. (1991). Single-particle tracking - analysis of diffusion and flow in 2-dimensional systems. Biophys. J.

[74] Sako Y., Kusumi A. (1994). Compartmentalized structure of the plasma-membrane for receptor movements as revealed by a nanometer-level motion analysis. J. Cell Biol.

[75] Sako Y., Kusumi A. (1995). Barriers for lateral diffusion of transferrin receptor in the plasma-membrane as characterized by receptor dragging by laser tweezers - fence versus tether. J. Cell Biol.

[76] Fujiwara T., Ritchie K., Murakoshi H., Jacobson K., Kusumi A. (2002). Phospholipids undergo hop diffusion in compartmentalized cell membrane. J. Cell Biol.

[77] Kusumi A., Nakada C., Ritchie K., Murase K., Suzuki K., Murakoshi H., Kasai R.S., Kondo J., Fujiwara T. (2005). Paradigm shift of the plasma membrane concept from the two-dimensional continuum fluid to the partitioned fluid: High-speed single-molecule tracking of membrane molecules. Annu. Rev. Biophys. Biomol. Struct.

[78] Iino R., Koyama I., Kusumi A. (2001). Single molecule imaging of green fluorescent proteins in living cells: E-cadherin forms oligomers on the free cell surface. Biophys. J.

[79] Morone N., Fujiwara T., Murase K., Kasai R.S., Ike H., Yuasa S., Usukura J., Kusumi A. (2006). Three-dimensional reconstruction of the membrane skeleton at the plasma membrane interface by electron tomography. J. Cell Biol.

[80] Suzuki K.G.N., Fujiwara T.K., Edidin M., Kusumi A. (2007). Dynamic recruitment of phospholipase C gamma at transiently immobilized GPI- anchored receptor clusters induces IP3-Ca2+ signaling: single-molecule tracking study 2. J. Cell Biol.

[81] Suzuki K.G.N., Fujiwara T.K., Sanematsu F., Iino R., Edidin M., Kusumi A. (2007). GPI-anchored receptor clusters transiently recruit Lyn and G alpha for temporary cluster immobilization and Lyn activation: single-molecule tracking study 1. J. Cell Biol.

[82] Ruan G., Agrawal A., Marcus A.I., Nie S. (2007). Imaging and tracking of tat peptide-conjugated quantum dots in living cells: New insights into nanoparticle uptake, intracellular transport, and vesicle shedding. J. Am. Chem. Soc.

[83] Chen B., Liu Q.L., Zhang Y.L., Xu L., Fang X.H. (2008). Transmembrane delivery of the cell-penetrating peptide conjugated semiconductor quantum dots. Langmuir.

[84] Ryser H.J.P., Hancock R. (1965). Histones and basic polyamino acids stimulate uptake of albumin by tumor cells in culture. Science.

[85] Zhao M., Kircher M.F., Josephson L., Weissleder R. (2002). Differential conjugation of tat peptide to superparamagnetic nanoparticles and its effect on cellular uptake. Bioconjug. Chem.

[86] Michalet X., Pinaud F.F., Bentolila L.A., Tsay J.M., Doose S., Li J.J., Sundaresan G., Wu A.M., Gambhir S.S., Weiss S. (2005). Quantum dots for live cells, in vivo imaging, and diagnostics. Science.

[87] Efros A.L., Rosen M. (1997). Random telegraph signal in the photoluminescence intensity of a single quantum dot. Phys. Rev. Lett.

[88] Frantsuzov P., Kuno M., Janko B., Marcus R.A. (2008). Universal emission intermittency in quantum dots, nanorods and nanowires. Nat. Phys.

[89] Kuno M., Fromm D.P., Hamann H.F., Gallagher A., Nesbitt D.J. (2001). “On”/“off” fluorescence intermittency of single semiconductor quantum dots. J. Chem. Phys.

[90] Shimizu K.T., Neuhauser R.G., Leatherdale C.A., Empedocles S.A., Woo W.K., Bawendi M.G. (2001). Blinking statistics in single semiconductor nanocrystal quantum dots. Phys. Rev. B.

[91] Wang X.Y., Ren X.F., Kahen K., Hahn M.A., Rajeswaran M., Maccagnano-Zacher S., Silcox J., Cragg G.E., Efros A.L., Krauss T.D. (2009). Non-blinking semiconductor nanocrystals. Nature.

